# The Association of Physical Activity and Sedentary Behaviors with Upper Respiratory Tract Infections and Sleep Duration in Preschool Children—Study Protocol

**DOI:** 10.3390/ijerph16091496

**Published:** 2019-04-27

**Authors:** Katarzyna Ostrzyżek-Przeździecka, Cynthia Smeding, Michał Bronikowski, Mariusz Panczyk, Wojciech Feleszko

**Affiliations:** 1 Department of Pediatric Pulmonology and Allergy, Medical University of Warsaw, Żwirki i Wigury 63A, 02-091 Warszawa, Poland; ostrzyzek.katarzyna@gmail.com (K.O.-P.); cynthia.smeding@gmail.com (C.S.); 2 Department of Didactics of Physical Activity, Poznan University of Physical Education, Królowej Jadwigi 27/39, 61-871 Poznań, Poland; bronikowski.michal@wp.pl; 3 Department of Education and Research in Health Sciences, Medical University of Warsaw, Żwirki i Wigury 81, 02-091 Warszawa, Poland; mariusz.panczyk@wum.edu.pl

**Keywords:** upper respiratory infections, pre-school children, physical activity, immune function, sedentary behaviors, sleeping habits

## Abstract

Currently, there is no consensus regarding the benefits of physical activity in terms of upper respiratory tract infections (URTIs) among different age groups of children. The number of school students avoiding physical education is on the rise. Children of all ages spend more time on sedentary behavior, eat less nutritious food and spend less time sleeping. All of these concomitant aspects adversely affect the immune system. A coexisting problem of a growing society is a large number of URTIs which is the main reason for general practitioner intervention. The aim of this study is to determine whether there is a correlation between the frequency of respiratory tract infections and the level of physical exercise in a cohort of pre-school children. This will be a cross-sectional, short-term study conducted on a single study population. We aim to recruit four-, to seven-year-old children who will be receiving activity monitoring devices for 24 h a day for 40 days. Daily step count, mean intensity of physical exercise and sleep duration will be measured. Simultaneously, their parents will receive a series of 60 questionnaires, one questionnaire per day, for the daily assessment of upper respiratory infection (URI) symptoms. Our study conducted on a cohort of healthy pre-school children using uniform tools, aims to scientifically establish and quantify the relationship between physical activity and health outcomes over a specified period of time.

## 1. Introduction

Amid concerns that increased sedentary behavior takes an increasing toll on the quality of life, there is also a growing debate as to the adverse effects on children and adolescents. The WHO [1] recommends that children and adolescents (aged 5–17) should participate in at least 60 min of moderate to vigorous-intensity physical activity (MVPA) daily. Tudor-Locke et al. analyzed reports on pre-school children. The analysis showed that 60 min of MVPA in children (aged 6–11 years) can be achieved with a mean 13,000–15,000 steps per day in boys and a mean of 11,000–12,000 steps per day in girls. [2] Limited evidence suggests that a total daily physical activity volume of 10,000–14,000 steps/day is associated with 60–100 min of MVPA in group of 4–6 years of age. Kantanista et al. reported that only 15.6% percent of girls in primary school achieved the recommended level of 12,000 steps per day [3].

Tudor-Locke et al. analyzed the reports on pre-school children [2]. Furthermore, Tudor-Locke and Bassett [4] established that the pedometer can be used to establish physical activity intervals (sex-specific) in children aged between 6–12 years. Particular physical activity categories are presented in the Table 1.

Nowadays, there is consensus that participation in physical activity, such as physical education at school, is precipitously declining [5]. Children of all ages spend more time on sedentary electronically-based activities (watching television, playing computer or video games, etc.), while sleeping less. It is estimated that six-year-old children and younger spend on an average two hours a day watching television, secondary to playing and outdoor activities.

Moderate physical activity (PA) has beneficial effects on the general health status and significantly reduces the risk of various diseases [6,7,8]. There is also evidence that health-related activity level during childhood has an impact on the risk of cardiovascular disease later on in life [9]. On the other hand, sedentary behaviors defined as “activities that do not increase energy expenditure substantially above the resting level and includes activities such as sleeping, sitting, lying down, and watching television, and other forms of screen-based entertainment” [10] have an influence on sleeping habits, patterns and general quality of life. Alarmingly, one third of 1–14-year-old Italian children sleep less than recommended and similarly one half of teenagers are sleep-deprived [11]. Modifiable risk factors for sleep deprivation and anomalies, such as the use of video devices, bedroom television, should be conspicuously curtailed as part of a corrective strategy. A systematic review by Wu et al. showed that the higher the frequency of physical activity, the less time is being spent on sedentary behaviour. Using data from cross-sectional and experimental studies Tudor-Locke et al. [4] provide evidence to support the use of low step counts to indicate a sedentary lifestyle (i.e., one characterized by more sedentary behavior and less ambulatory behavior) in adults. These findings suggest that health programs at school promote active lifestyles among children and adolescents and may contribute to the overall enhancement of quality of life [12]. A cross-sectional research survey conducted on a cohort of children between 8–11 years of age (*N* = 826) indicated that electronic screen time (ST) was associated with an increased risk of obesity regardless of physical activity [13].

On a multi-national scale, URIs, including the common cold and influenza, are the most commonly quoted causes for individuals of all ages to visit a general practitioner. Preschool children are particularly prone to recurrent respiratory infections, mostly respiratory infections [14]. Therefore, there is an on-going endeavor to comprehensively mitigate these types of infections and to implement strategies to reduce their occurrence [15]. Several studies also extrapolate a negative correlation between immune function and prevalent upper respiratory tract infections. Conversely, studies on adult populations [7,16,17,18,19], indicate that regular PA carries significant benefits in terms of incidence and severity of URIs. Nevertheless, only a few studies have heretofore been published that address this relationship within the pediatric population. Data on study groups including children between seven and 14 years of age [20,21,22,23] did not necessarily support a clear conclusion that PA actually mitigates recurrent respiratory infections. Canadian mechanistic research indicated that children, who spent more time on sport activities and aerobic fitness activities, reported fewer “sick” leaves [20]. However, an ambiguous picture emerges from studies conducted among young athletes. Moreira et al. [24] which showed a significant increase in immunoglobulin A (sIgA) secretion and reduced URTIs in athletes abstaining from physical training after a two-week period. In contrast, an observational study on a basketball player cohort showed that an increased training load actually had a negative effect on the immune function of the respiratory mucosa, but showed no significant impact on the severity of URTIs [25]. Conversely, Osterback et al. observed that active participation in sports bore no benefits on recurrent respiratory diseases [26].

Due to the ubiquitous nature of these infections, a systematic literature review raised awareness of the urgent need for methodologically and scientifically rigorous studies to extrapolate the relationship between PA and infections in pre-school children, who are particularly susceptible to URTIs. It was proven that to reap health benefits, children and adolescents should participate in moderate-to-vigorous PA one or more hours per day [8,27,28], but its impact on the respiratory immunity remains elusive.

More extensive data collection utilizing standardized tools as well as scientific findings could provide a baseline for developing guidelines and versatile intervention protocols for boosting the immune system, with tangible public health benefits. Enhanced efficacy of such guidelines and protocols could be achieved by seamlessly incorporating immune-boosting exercise programs that are interwoven into a daily routine of pre-school children.

To meet the aforementioned challenge, we have conceived a protocol for an observational study using uniform tools for the assessment of study parameters, aiming to obtain the most reliable data thereby yielding a verification of our working hypothesis.

The aim of this study is to determine whether there is a relationship between active lifestyle, particularly the level of PA, expressed as the average daily step count, and recurrent URIs in pre-school children. The ultimate goal of this investigation will be to develop guidelines for PA in pre-school children, in an effort to forestall recurrent URTIs. Our research hypothesis assumes that more active children, who have a higher average daily step count, are less likely to present with symptoms of URIs.

## 2. Material and Methods

### 2.1. Study Design

This will be a cross-sectional, short-term study conducted on a single study population. The research will be conducted and supervised by the Department of Pediatric Pneumology and Allergology of the Teaching Hospital of the Medical University of Warsaw.

### 2.2. Study Population

Our study included pre-school children between four and seven years of age, comprising both male and female individuals who regularly attend pre-school. There is a particular risk of developing recurrent respiratory infections in this age group due to a constant pathogen exposure among peers, different anatomy of the respiratory system and an immature immune system. Furthermore, since early childhood is a pivotal period for the initiation of a healthy lifestyle, it seems imperative that research be conducted on the association between PA and respiratory health of pre-schoolers [29].

#### Inclusion/Exclusion Criteria

Children meeting the following criteria will be included in the study:

- 4–7 years of age (48 months–6 years and 11 months)
- regular pre-school attendance
- no contra-indications for PA

Exclusion criteria:

- Known contra-indications for PA determined upon study onset
- Immune deficiency, chronic severe respiratory diseases (cystic fibrosis, interstitial disease, hemosiderosis, asthma)
- Known allergies causing upper and lower respiratory symptoms (allergy to animal fur, dust, fungi, dust mites, grass and tree pollen)
- Lack of consent from parent or legal guardian

### 2.3. Procedure and Observation

At the beginning of the autumn/winter season, parents of pre-school children will be provided information on the research at the facility, the purpose of which will be to assess the relationship between the level of PA and the rate of recurrent respiratory infections. Parents willing to enroll their children in the study will receive a two-fold questionnaire package comprising a qualification and a personal questionnaire. The former will establish qualification criteria whereas the latter will go into depth on issues such as PA, health status and risk factors likely to affect this study. Data from the questionnaires will provide the basis for establishing a research group. This questionnaire was devised by the authors specifically for the study.

**Figure 1:**
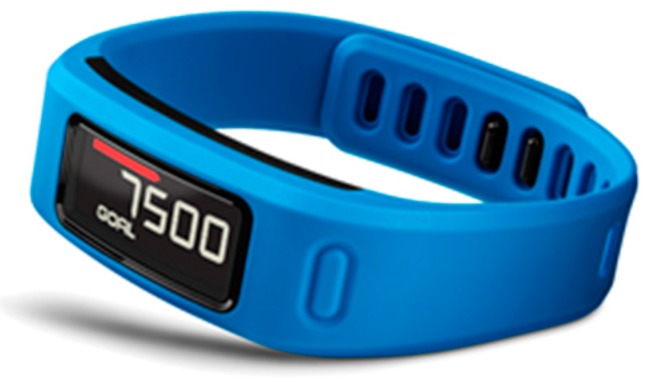
Garmin Vivofit 1—monitoring band [34].

At baseline, the qualified children will receive activity monitoring devices to measure their daily step count, mean intensity of physical exercise and sleep duration. Thus far, only few studies have been published using accelerometers for the measurement of PA in pre-school children. All these studies showed that this kind of device is safe and appropriate for this age group [30,31,32,33]. We chose Garmin Vivofit^®^ fitness band (Garmin Ltd., Olathe Kansas, US) [34] (Figure 1) with a weight of 25.5 g and a size of 21 mm (width) × 10.5 mm (thickness). Each child will wear the band 24 h a day for 40 days. Participants will be allowed to remove the band during bath time and occasionally to assess the skin for potential intertrigo or allergies.

**Figure 2:**
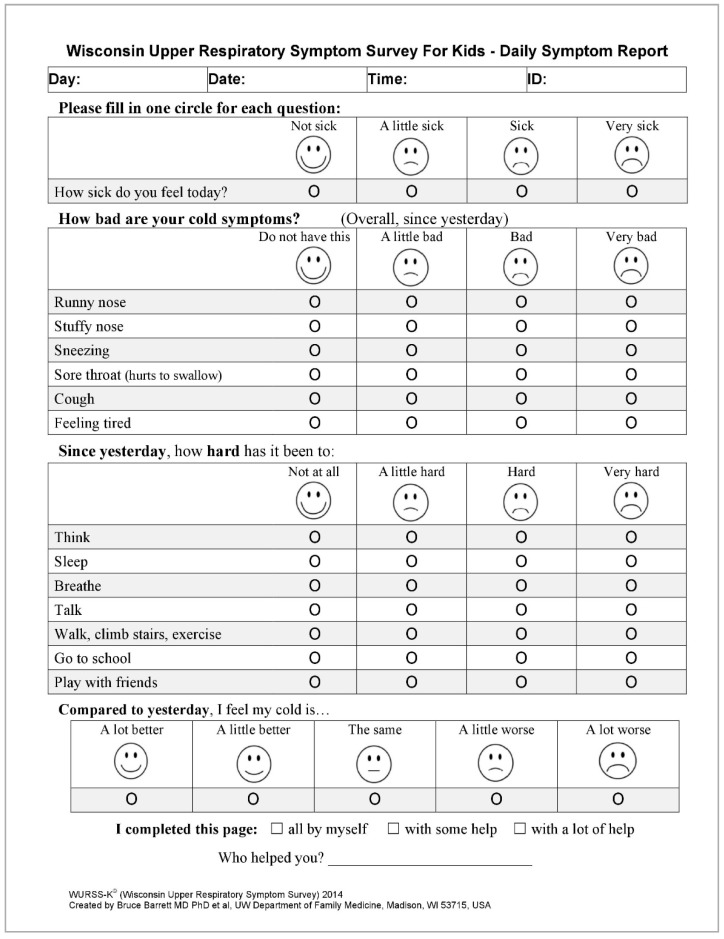
Wisconsin Upper Respiratory System Survey for Kids (WURSS-K)—questionnaire for daily assessment of the symptoms of URIs [35].

Simultaneously, parents/guardians will receive a set of 60 questionnaires, one questionnaire per day, for the daily assessment of URI symptoms. The questionnaire is a Polish version of the Wisconsin Upper Respiratory System Survey for Kids (WURSS-K), which is currently being validated [35]. It contains a set of questions for the assessment of the presence and the severity of URTIs and their effect on the quality of life. The questionnaire consists of 4 questions (Figure 2). The 1st question refers to the state of well-being: “How sick do you feel today?”. The second question refers to the severity of different symptoms, such as rhinitis, nasal obstruction, sneezing, sore throat, cough, and fatigue. In the third question, subjects are required to assess their difficulty in: thinking, sleeping, breathing, speaking, walking in general, walking stairs, exercising, attending school/pre-school, and playing after presenting with symptoms the day before. The fourth question refers to the assessment of common cold symptoms compared with the day before. One of the four answers may be selected for each question to best describe the current state. In order to facilitate the choice, each answer is accompanied by a picture of a face reflecting the subject’s mood.

Each day for 60 days, the subject, assisted by a parent will complete the questionnaire. As far as the questionnaire is concerned, the role of the parent is to clearly read the questions and relate the corresponding choice of answers to the child. The child is then supposed to mark the appropriate box representing the selected answer.

### 2.4. Outcome Measures

Physical activity monitoring bands deliver the following data:

- Daily step count
- Daily percentage in proportion to different exercise intensities (low, medium, high)
- Daily sleep duration

Data from the questionnaires enquiring about the symptoms of URIs will allow for the calculation of the following parameters:

- Total number of days with the symptoms of URTI
- Infection severity expressed as a sum of scores
- The most predominant symptoms
- The severity of different symptoms.

Data on the child’s exposure to 2nd hand tobacco smoke, the presence of animal hair allergens, sleep duration (as reported by parents), passive activity duration (electronic ST) and the age of the child will be collected from qualification questionnaires completed by parents at baseline. Once the qualification process is over, all study participants will be weighed and measured by the same investigator to obtain uniform data for body mass index (BMI) and Cole’s index calculation.

### 2.5. Endpoints

For the estimation of the relationships between the measured PA and the questionnaire data on URIs, the following parameters will be compared:

- PA (expressed as an average daily number of steps)
- BMI
- sleep duration (both reported by parents and objectively measured by the monitoring band)
- passive activity duration (as reported by parents)
- Second hand tobacco smoke exposure
- allergen exposure in the form of animal fur, and the total number of days with the symptoms of URIs, the sum of scores for the symptoms assessed in the questionnaire.

### 2.6. Contact with Other Participants

Information on the research conducted at the pre-school facility will be provided to parents in the form of leaflets and during individual discussions with a research group representative. The investigator will be in constant telephone and e-mail contact with parents from the 1st day of the study (i.e., the day when physical activity monitoring devices and a set of questionnaires will be provided). Parents will be informed about the day of data extraction from monitoring devices via text messages or e-mails one day in advance. If the child is absent on a particular day, data extraction will be re-scheduled individually. To avert inconsistencies that may occur in the completion of the symptom questionnaire, a reminder message will be sent every day in the afternoon and evening hours to alleviate data gaps. Regular personal contact with the study participants (both with child and parent), at least once a week, will allow for quick detection and correction of errors in the observation, monitoring equipment functioning as well as an evaluation of participant motivation to continue the study.

### 2.7. Criteria Preventing Study Continuation

Participants who due to health issues will no longer be able to undertake daily PA (immobilization, long-term hospital stay), who lose their monitoring devices, or voluntarily withdraw from the study will be excluded.

### 2.8. Sample Size

The research is to be conducted at pre-schools in the Warsaw city region. We aim at a research target population which is comprised of children between 4–7 years of age from the Mazowieckie voivodeship in Poland. According to the 2018 Demographic Yearbook of Poland by Statistics Poland [36] the target research group is estimated to be 231,608 individuals. Unfortunately, Statistics Poland does not provide a detailed breakdown by age groups for individual voivodeships. Thus, the research data estimates are based on tables: (1) People in Poland by sex and age, and (2) People in Poland by age and voivodeships [36].

Simple Size Calculator [37] was used to compute the number of individuals in the target research study cohort. The following assumptions were used: Confidence Level 95%, proportion 0.5, Confidence Interval 0.1, and Standard Error 0.05. It was also assumed that about 10% of the outcomes can potentially be eliminated from the study as ‘incomplete’. Finally our study selected at a final cohort of about 110 participants.

### 2.9. Enrolment

Enrollment will be performed at three pre-schools (two public and one private) in the Warsaw city region. All parents of children between four and seven years of age will receive information about the study and a qualification questionnaire, the completion of which will be (along with providing contact details) the first step in the enrollment process. All activities performed at the facility will be conducted with consent and full school management approval.

### 2.10. Data Collection and Management

All data obtained from qualification questionnaires will be saved in individual Microsoft Office Excel (Microsoft Corporation, Redmond, Washington, U.S.) spreadsheets. Each participant will be given an identification number corresponding to the serial number of the activity monitor to secure personal data.

Starting from baseline, the data collected in the activity monitoring device worn by the subject will be copied to the computer’s memory every seven days. A computer application known as Garmin Express^®^ (Garmin Ltd., Olathe, Kansas, U.S.) will be used to facilitate the configuration, registration and management of all monitoring devices. This will be performed at the pre-school facility during working hours. In case of a subject’s absence, an individual appointment with the parent will be scheduled for data extraction. During the synchronization check, the battery status will be assessed. Extensive memory of the device will allow for up to a three-week interval in data extraction, which, considering a weekly synchronization, excludes the possibility of data loss even in the case of a few-days absence.

Collected data will be evaluated using the Garmin Connect^®^ (Garmin Ltd., Olathe, Kansas, U.S.) by logging into individual accounts of the study participants. The collection of questionnaires regarding the symptoms of URIs will be conducted in two stages. At baseline, each parent will receive two sets of questionnaires, 30 questions each. After the first of the 30 days, the subject’s parents or guardians will receive a text message asking them to leave the completed questionnaires at the facility. The same procedure will be repeated the following month. Data from the questionnaires will be transcribed instantly to specially designed Microsoft Office Excel sheets, where qualitative data will be replaced with continuous variables accordingly, as per previously prepared instructions. In addition, physical activity data will be recorded in the aforementioned program, creating a comprehensive and complete database along with the remaining Excel sheets.

### 2.11. Statistical Analysis

The Statistica (Dell, Inc., Round Rock, Texas, U.S) software package will be used for statistical analysis. Descriptive statistics will be utilized to summarize the basic features and develop the characteristics for the study group. The Pearson correlation coefficient will be used to assess the correlation between the average number of steps (in accordance with recommendations [2,38]) and the number of days with symptoms of infection, the sum of scores for the symptoms of infection, and the sum of scores for the state of well-being (feeling ill). Goodman and Kruskal’s lambda coefficient (λ) will be used to measure the strength of the relationship between ordinal variables: sleep duration length (four groups) and the number of days with symptoms of infection as well as passive activity (four groups) and the number of days with symptoms of infection. In order to categorize participants into three research cohorts differing in the level of PA, we will use K-means clustering, imposing a priori division of the study group into three subgroups. All statistical hypotheses will be verified with an assumed significance level of 5%.

### 2.12. Missing Data

Data gaps that may arise in the questionnaire regarding symptoms of infection will be supplemented with the results averaged from the values entered in the questionnaire on the preceding or the following day, if these concern only single days. The minimum value of completed questionnaires is assumed to be 54. Participants submitting seven or more blank questionnaires will be excluded from the statistical analysis. A daily number of steps below 1000 will also be considered as missing data and will be supplemented as previously described above. The minimum number of days with correctly counted steps is 36.

### 2.13. Ethics and Popularization

#### 2.13.1. Ethics and Consents

Our study was approved by the Bioethical Commission of the Medical University of Warsaw. Initially, parents or legal guardians who verbally consented to the participation of their child in the study will receive participant information sheets containing all pertinent details regarding the aim and the course of the study, possible inconveniences related to participation, as well as information about the possibility of withdrawal at will. Parents will sign an informed participation consent form prepared in accordance with the guidelines of the Bioethical Commission on the first day of the study. Any modifications in the protocol that may affect the course of the study, the potential benefits to patients, patient safety, including changes in the study design, will be reported to the Bioethical Commission prior to introducing all changes. All personal information related to the study will be encrypted by registration number and securely stored on a stand-alone, password secured computer. The Bioethical Commission does not allow financial rewards for subjects who participate in a study. Nevertheless, the parents will get an individual analysis of their child’s results in comparison to the study cohort and an individual assessment of physical activity for future prevention of recurrent respiratory infections.

#### 2.13.2. Popularization Plan

The manuscript encompassing our research results will be submitted for publication, peer-reviewed, and will be presented at local and international medical conferences. Our findings will also be made available to participants in the form of a summary and an individual assessment of each child compared to the study cohort. Furthermore, this study will be the subject of a PhD thesis (K.O.-P).

## 3. Discussion

It is currently under debate what intrinsic and/or extrinsic environmental factors moderate immunity in children or adults, subsequently influencing the frequency of URTI [23]. Thus far, a few studies have been published regarding the relationship between the level of PA and the frequency of recurrent infections of the respiratory system in children. Furthermore, the results of the published studies are not mutually consistent and the conclusion leads to the need for further research.

Undoubtedly, it is necessary to create a research protocol approach to evidence based medicine, using objective measurement tools and minimizing possible measurement errors and biases.

Moderate levels of physical activity have been associated with a lower incidence of URTI in children, whereas adolescents who spent less time in sport activities reported a significantly higher frequency of URTI [21,22]. However, both of this these studies have used only subjective measures of PA, which are likely to misrepresent actual levels of physical activity. Jedrychowski et al. [22] qualified 1028 children attending to the second-classes of primary school. PA level was categorized by the answers from questioners. In scoring the physical activity level, data on regular exercise and the number of daily hours spent watching TV or doing homework have been used. Klentrou et al. and Cieslak et al. [20,21] used the Habitual Activity Estimation Scale (HAES) to estimate the duration of all forms of habitual activity. Predicted peak aerobic power was estimated using the 20-m shuttle run of Leger and Lambert. What is more in one of this studies [21] the Participation Questionnaire (PQ) was used to estimate both the amount of participation in physical activity and the nature of the participation in three categories: free time activity (FTA), organized activity time (OAT), and total time spent in activities (SAT). The research evaluative discussed in 10- to 11-year-old children attending public schools in Southern Ontario [20] using objective methods to measure daily distance traveled. Digi-Walker pedometer (NewLifestyles) recorded the child’s physical activity in steps by using a step counter. All subjects were required to use the Digi-Walker for two consecutive days. Each monitor was calibrated to accurately record the movements of the subject. A two-day activity log accompanied the pedometer. The subjects also recorded daily physical activities other than general locomotion. In contrast, the Corbett study [23] quantified physical activity by using Actigraph GT1M accelerometers. Participants were instructed to wear the accelerometer on the right hip from the moment they woke up until they went to sleep for seven days starting the day after their visit to the laboratory.

Simultaneously, in all of these studies, self-reported methods were used to report the incidence of URTIs. A one-month health log [39] was used to record the incidence and duration (number of days) of URTIs [20,21,23]. Subjects recorded cold and flu symptoms each day of the month by using a set of codes provided with the log. The severity of the symptoms was rated by each subject as mild, moderate, or severe. The total number of days with URTI symptoms was calculated for each subject, with days being counted only if two or more consecutive days of cold or flu symptoms were reported. Jedrychowski et al. used the health survey that provided data on the number of respiratory infections that took place in the two past years. Recurrent acute respiratory infections were defined as 10 or more episodes experienced by the child over a two-year follow up [22]. Despite the fact that all these studies have been carried out in groups of children, none of them concerned pre-school children. For this reason, the preparation of the above study protocol was a great challenge for us. We believe that the research methodology and tools we propose will reduce trial bias.

## 4. Conclusions

There is some wide-ranging perplexity and dialogue regarding a potential correlation between the incidence of recurrent respiratory infections (defined as three or more infections per fall-winter season [40]) and physical activity in pre-school children. No conclusive answer has yet been found so far. Our study, conducted among a population of healthy pre-school children using objective tools measuring PA, aims to scientifically establish and quantify the relationship between PA and health outcomes over a specified period of time. The negative impact of PA, or rather the lack thereof, on sleep duration and its quality has been well documented. It may well be that sleep duration is associated with URI frequency.

If the research hypothesis is confirmed, a subsequent experimental interventional study is planned in order to determine causal relationships as well as to verify which factor in particular, i.e., PA or the incidence of URTIs, is either a dependent or an independent variable. Our underlining preliminary assumption is that sleep duration is an indirect factor, PA is an independent variable, whereas the incidence of URTIs is a dependent variable.

## Figures and Tables

**Table 1:** Physical activity categories and sex-specific intervals for children (ages 6–12 years) [4].

Physical Activity Categories	Boys	Girls
Sedentary	<10,000	<7000
Low active	10,000–12,499	7000–9499
Somewhat active	12,500–14,999	9500–11,999
Active	15,000–17,499	12,000–14,499
Highly active	≥17,500	≥14,500
